# Is There an Association between Post-Traumatic Stress Disorder and the Incidence of Chronic Low Back Pain?

**DOI:** 10.3390/jcm12175753

**Published:** 2023-09-04

**Authors:** Karel Kostev, Lee Smith, Josep Maria Haro, Marcel Konrad, Ai Koyanagi, Louis Jacob

**Affiliations:** 1 Epidemiology, IQVIA, 60549 Frankfurt, Germany; 2 University Clinic of Philipps University, 35037 Marburg, Germany; 3 Centre for Health, Performance and Wellbeing, Anglia Ruskin University, Cambridge CB1 1PT, UK; 4 Research and Development Unit, Parc Sanitari Sant Joan de Déu, CIBERSAM, ISCIII, Dr. Antoni Pujadas, 42, Sant Boi de Llobregat, 08830 Barcelona, Spain; 5 Health & Social, FOM University of Applied Sciences for Economics and Management, 45127 Frankfurt am Main, Germany; 6 Université Paris Cité, AP-HP, Lariboisière-Fernand Widal Hospital, Physical Medicine and Rehabilitation Department, 75010 Paris, France; 7 Université Paris Cité, Inserm U1153, Epidemiology of Ageing and Neurodegenerative Diseases, 75010 Paris, France

**Keywords:** post-traumatic stress disorder, low back pain, Germany, cohort study, epidemiology

## Abstract

Background: Preliminary research suggests post-traumatic stress disorder (PTSD) is a risk factor for chronic low back pain (CLBP). However, this literature displays some limitations. Therefore, this study aimed to investigate the association between PTSD and the 10-year cumulative incidence of CLBP in adults from Germany. Methods: The present retrospective cohort study included adults diagnosed with PTSD in 1 of 1284 general practices in Germany in 2005–2020 (index date). Individuals without PTSD were matched to those with PTSD (1:1) using a propensity score based on age, sex, index year, duration of follow-up, and the mean number of consultations during follow-up. In patients without PTSD, the index date was a randomly selected visit date. Results: There were 60,664 patients included in the study. After adjusting for frequent comorbidities, there was a positive but non-significant association between PTSD and incident CLBP in the overall population (HR = 1.07, 95% CI = 0.99–1.15). Nonetheless, the relationship between PTSD and CLBP was statistically significant in the age group >60 years (HR = 1.24, 95% CI = 1.05–1.46). Conclusions: Conversely to previous research, PTSD was not associated with incident CLBP in this large German sample. Further longitudinal studies are warranted to corroborate these findings before drawing any firm conclusions.

## 1. Introduction

Chronic low back pain (CLBP) is defined as low back pain lasting more than three months [1]. The prevalence of CLBP is high in the general population and reaches 20% in working-age adults [2]. Around 10% of patients with acute low back pain will develop CLBP [3]. CLBP is a risk factor for impaired quality of life [4], disability [5], and several physical conditions (e.g., diabetes [6], coronary heart disease [7], and stroke [8]). In addition, CLBP has deleterious effects on work participation [9] and is associated with a major economic burden for patients and healthcare systems [10]. In this context, identifying risk factors for CLBP is a public health priority.

There is a strong relationship between mental health and CLBP. CLBP is positively associated with the future onset of multiple psychiatric conditions, such as anxiety disorder and depression [11,12]. Poor mental health has also been found to predict the incidence of low back pain. For example, a systematic review and meta-analysis of 19 cohort studies identified a positive and significant association between depressive symptoms and the risk of low back pain [13]. Interestingly, other research showed that post-traumatic stress disorder (PTSD) is a risk factor for future CLBP [14,15,16,17]. PTSD occurs after exposure to a threatening or horrific event (e.g., combat, natural disaster, and witnessing the sudden death of others) and corresponds to symptoms (e.g., re-experiencing the traumatic event, deliberate avoidance of reminders, and persistent perceptions of heightened current threat) lasting at least several weeks [18]. The effects of PTSD on CLBP could be mediated by multiple factors, such as low-grade inflammation [19,20], insufficient physical activity [21,22], smoking [23,24], and alcohol consumption [25,26]. Although the previous literature has advanced the field, this research displays some limitations that should be acknowledged. First, half of the studies included men only and had small sample sizes (i.e., less than 1000 participants) [15,17], limiting the generalizability of the findings. Second, data were mostly collected in the United States of America [14,15,17], and thus, little is known about the association between PTSD and CLBP in other countries. Third, no repeated measures were available for CLBP for most studies, and it was not possible to analyze the effects of PTSD on the incidence of CLBP [14,15,16]. Fourth, and more notably, all analyses were unadjusted for other chronic conditions [14,15,16,17], even though some of these disorders may play a confounding role (e.g., obesity [27,28], diabetes mellitus [29,30], and sleep disorders [31,32]). Taking these limitations together, more research is warranted on the relationship between PTSD and CLBP.

Therefore, the aim of this study was to investigate the association between PTSD and the 10-year cumulative incidence of CLBP in adults followed in general practices in Germany. Given that the present research included men and women, had a large sample size, was of a longitudinal nature, and included frequent comorbidities, the study overcomes most of the limitations mentioned above.

## 2. Methods

### 2.1. Ethics Approval and Consent to Participate

German law allows the use of anonymous electronic medical data for research under certain conditions. Given this legislation, these deidentified records can be used without obtaining written informed consent from the patients and approval from a medical ethics committee.

### 2.2. Database

Data from the Disease Analyzer database (IQVIA) were used for the present study. The methodology of this database has already been described in the literature [33]. Briefly, the Disease Analyzer database contains demographic, diagnosis, and prescription data from general and specialized practices in Germany. Diagnoses are coded using the International Classification of Diseases, 10th revision (ICD-10), while prescriptions are coded using the Anatomical Classification of Pharmaceutical Products of the European Pharmaceutical Market Research Association (EphMRA). The data are collected every month in an anonymous format from the computer systems of the practices, and the quality of the data is assessed based on several criteria (e.g., completeness of information and linkage between diagnoses and prescriptions). The selection of practices to include in the database relies on multiple variables, such as physician’s age, specialty group, community size category, and German federal state. Finally, the Disease Analyzer database includes 3% of all practices in Germany and has been found to be representative of these practices [33].

### 2.3. Population

**Figure 1:**
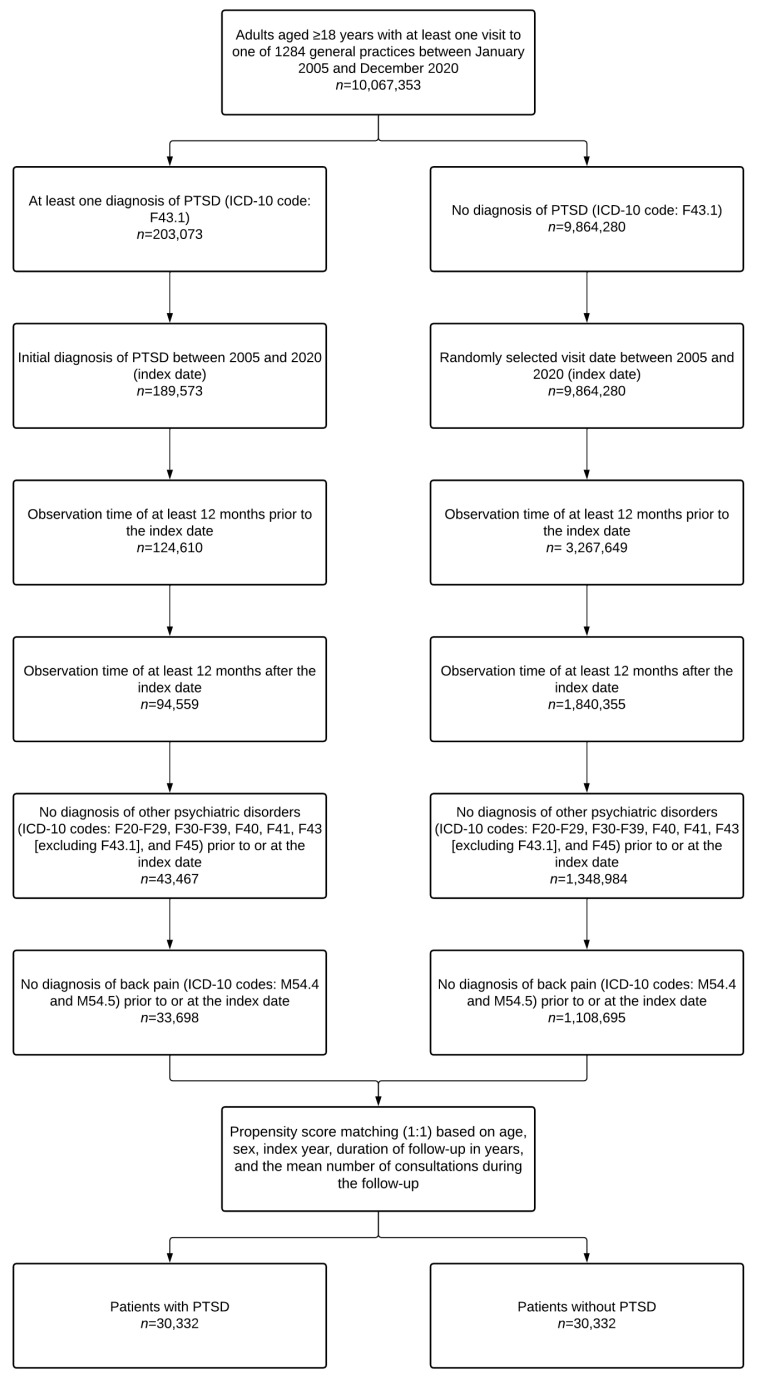
Selection of study patients. Abbreviations: PTSD, post-traumatic stress disorder; ICD-10, International Classification of Diseases, 10th revision.

The present research was exploratory and did not require calculating a sample size. This retrospective cohort study included all adults aged ≥18 years who were diagnosed for the first time with PTSD (ICD-10 code: F43.1) in 1 of 1284 general practices in Germany between January 2005 and December 2020 (index date). Other inclusion criteria included observation time of at least 12 months prior to the index date; observation time of at least 12 months after the index date; no diagnosis of other psychiatric disorders (ICD-10 codes: F20-F29, F30-F39, F40, F41, F43 (excluding F43.1), and F45) prior to or at the index date; and no diagnosis of back pain (ICD-10 codes: M54.4 and M54.5) prior to or at the index date. After applying similar inclusion criteria, individuals without PTSD were matched (1:1) to those with PTSD using a propensity score based on age, sex, index year, duration of follow-up (in years), and the mean number of consultations during the follow-up. In patients without PTSD, the index date was a randomly selected visit date between January 2005 and December 2020. The selection of study patients is displayed in Figure 1.

### 2.4. Outcome

The outcome of the study was the 10-year cumulative incidence of CLBP in individuals with and without PTSD. CLBP corresponded to the presence of low back pain (i.e., lumbago with sciatica (ICD-10 code: M54.4) or low back pain (ICD-10 code: M54.5)) at two different medical consultations with at least 90 days between them.

### 2.5. Covariates

Covariates included age at baseline, sex, duration of follow-up (in years), the mean number of consultations during the follow-up, and conditions documented in at least 5% of individuals with PTSD within 12 months prior to or at the index date. Conditions were hypertension (ICD-10 code: I10), disorders of the thyroid gland (ICD-10 codes: E00-E07), disorders of lipoprotein metabolism and other lipidemias (ICD-10 code: E78), gastritis and duodenitis (ICD-10 code: K29), injury of unspecified body region (ICD-10 code: T14), diabetes mellitus (ICD-10 codes: E10-E14), chronic sinusitis (ICD-10 code: J32), sleep disorders (ICD-10 code: G47), other and unspecified dermatitis (ICD-10 code: L30), joint disorders, not elsewhere classified (ICD-10 code: M25), gastro-esophageal reflux disease (ICD-10 code: K21), asthma (ICD-10 code: J45), overweight and obesity (ICD-10 code: E66), vasomotor and allergic rhinitis (ICD-10 code: J30), other enthesopathies (ICD-10 code: M77), migraine (ICD-10 code: G43), cancer (ICD-10 codes: C00-C97), chronic ischemic heart disease (ICD-10 code: I25), shoulder lesions (ICD-10 code: M75), other disorders of muscle (ICD-10 code: M62), and varicose veins of lower extremities (ICD-10 code: I83).

### 2.6. Statistical Analyses

Demographic (i.e., age and sex) and clinical characteristics (i.e., duration of follow-up, the mean number of consultations during the follow-up, and conditions documented within 12 months prior to or at the index date) were compared between adults with and those without PTSD using the Wilcoxon signed-rank test for continuous variables, the McNemar test for categorical variables with two categories, and the Stuart–Maxwell test for categorical variables with more than two categories. There were no missing data in the variables assessed before or at the index date. The 10-year cumulative incidence of CLBP in the PTSD and no-PTSD group was further studied using Kaplan–Meier curves, and comparisons were made based on the log-rank test. Loss to follow-up corresponded to a follow-up of fewer than 10 years, and it was hypothesized that loss to follow-up was unrelated to the occurrence of CLBP. Finally, Cox regression models were used to investigate the association between PTSD and incident CLBP in the overall sample and age (i.e., 18–30, 31–40, 41–50, 51–60, and >60 years) and sex subgroups (i.e., female and male). The models were adjusted for the comorbidities mentioned above but not age, sex, index year, duration of follow-up, or the mean number of consultations during the follow-up, as these variables were used to match individuals without PTSD to those with PTSD. Stratified analyses were justified owing to the fact that there are substantial age and sex differences in the epidemiology of CLBP [2]. The results of the Cox regression analyses are displayed as hazard ratios (HRs) and 95% confidence intervals (CIs). *p*-values lower than 0.050 were considered statistically significant. All analyses were conducted with SAS 9.4.

## 3. Results

There were 30,332 patients with and 30,332 patients without PTSD included in this retrospective cohort study. The mean (standard deviation) age was 46.0 (16.7) years in the PTSD group and 46.0 (16.8) years in the no-PTSD group, while there were 62.7% of women in both samples (Table 1).

**Figure 2:**
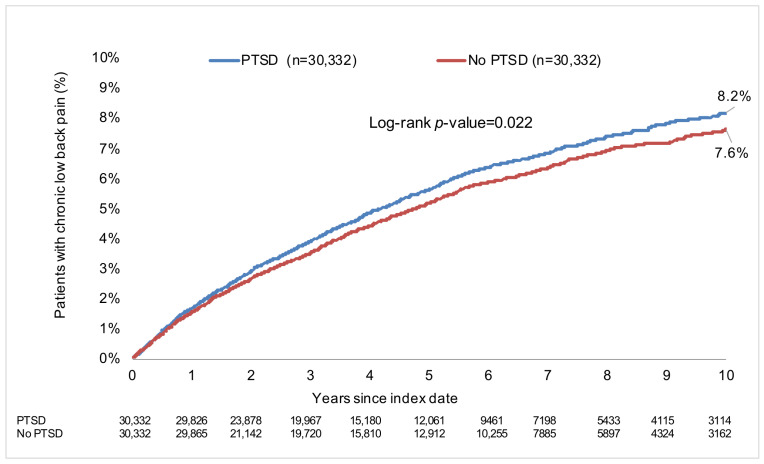
Ten-year cumulative incidence of chronic low back pain in people with and without post-traumatic stress disorder (Kaplan–Meier analysis). Abbreviation: PTSD, post-traumatic stress disorder. The two Kaplan–Meier curves were compared using the log-rank test.

The mean (standard deviation) duration of follow-up was 5.2 (3.9) and 5.7 (4.0) years in participants with and without PTSD, respectively. Hypertension (26.5% and 27.8% in the PTSD and no-PTSD group, respectively), disorders of the thyroid gland (23.0% and 23.1%), and disorders of lipoprotein metabolism and other lipidemias (18.8% and 17.2%) were the three most frequent conditions documented within 12 months prior to or at the index date. After 10 years of follow-up, 8.2% of people with PTSD and 7.6% of those without PTSD were diagnosed with CLBP (log-rank *p*-value = 0.022; Figure 2).

The results of the adjusted Cox regression models are displayed in Table 2. In the overall population, PTSD was positively associated with incident CLBP, but this relationship did not reach statistical significance (HR = 1.07, 95% CI = 0.99–1.15). Non-significant findings were obtained in the age- and sex-stratified analyses, except in the age group >60 years in which the association was statistically significant (HR = 1.24, 95% CI = 1.05–1.46).

## 4. Discussion

### 4.1. Main Findings

This retrospective cohort study, including more than 60,000 people from general practices in Germany, found that the 10-year cumulative incidence of CLBP was significantly higher in the PTSD than in the no-PTSD group (8.2% versus 7.6%). However, after adjusting for frequent comorbidities, the Cox regression model found a positive but non-significant association between PTSD and incident CLBP in the overall sample, with a narrow CI (HR = 1.07, 95% CI = 0.99–1.15). Interestingly, the relationship reached statistical significance in the age group >60 years. To the best of the authors’ knowledge, this is the largest study on the topic and the research with the most extensive follow-up.

### 4.2. Interpretation of Findings

A critical finding of this study is the lack of a statistically significant relationship between PTSD and the cumulative incidence of CLBP after adjusting for frequent comorbidities. This result is not in line with the scientific literature, and there are several hypotheses to explain this discrepancy. First, previous studies did not adjust for frequent chronic comorbidities, which may be confounding factors in the association between PTSD and CLBP. For example, obesity [27,28], diabetes mellitus [29,30], and sleep disorders [31,32] are associated with both PTSD and CLBP, and not adjusting for these disorders may have biased the previous estimates. Second, two of the studies relied on survey data, which were self-reported [16,17]. These self-reported data may be biased (e.g., recall bias) and do not necessarily correspond to diagnoses. Third, in this body of research, participants were most of the time evaluated at a single time or were followed for a limited amount of time [14,15,16], whereas the present study investigated the effects of PTSD on the incidence of CLBP using survival analyses based on repeated measures for CLBP. Fourth, in two studies, patients were followed in tertiary care centers [14,15]. These patients may have more severe PTSD symptoms than those from general practices included in this German study and may be more prone to developing low back pain.

Another interesting result is the positive and significant association between PTSD and incident CLBP in people aged >60 years. It is possible that PTSD has stronger deleterious effects on low back pain at older age [34]. Given that chronic pain is frequent in older adults [35], patients aged >60 years may be more likely to suffer from pain in another region of the body and be more vulnerable to the deleterious impact of PTSD on low back pain compared with their counterparts aged 18–60 years. Moreover, frailty is common in older people [36], and the occurrence of acute stressors may accelerate unintentional weight loss and decline in physical activity, which could favor the subsequent incidence of low back pain. Finally, coping with PTSD symptoms may be insufficient, and the association between PTSD and incident CLBP may be mediated by several unhealthy behaviors (e.g., smoking [23,24] and alcohol consumption [25,26]).

### 4.3. Clinical Implications and Directions for Future Research

In people aged >60 years, general practitioners should regularly assess low back pain in those who have experienced PTSD. Exercise and physical activity (e.g., resistance training, walking, and yoga) are critical non-pharmacological treatments for PTSD [37] and CLBP [38], which should be largely promoted. Some of these exercises can be specific and may prevent the occurrence of low back pain, such as core-strengthening and lumbar stabilizing exercises and postural training. In addition, in patients with moderate-to-severe PTSD symptoms, referral to mental health professionals (e.g., psychiatrists and psychologists) should be considered, and the early initiation of pharmacological treatments (e.g., selective serotonin reuptake inhibitors and serotonin-norepinephrine reuptake inhibitors) may be an option. In terms of future research, more longitudinal studies are needed to corroborate or invalidate the present findings. Data are also warranted to investigate the potential dose–response relationship between the intensity of PTSD symptoms and the risk of CLBP.

### 4.4. Strengths and Limitations

The major strengths of this study are the large sample size, the duration of follow-up, and the use of data collected in general practices. Nonetheless, the study findings should also be interpreted in the light of several limitations. First, diagnoses relied on ICD-10 codes only, and more information on PTSD (e.g., type of stressor) and CLBP (e.g., intensity) would have allowed more detailed analyses. Second, there was a lack of data on behavioral factors (e.g., smoking status and consumption of alcohol), although these factors may have impacted the cumulative incidence of PTSD. Third, the 10-year incidence of PTSD was slightly lower than previous figures obtained in primary care [39], and it is possible that this psychiatric disorder has been underdiagnosed. Fourth, PTSD and CLBP may have been diagnosed in specialized practices such as psychiatric and rheumatology practices, and the results of this study may not be extrapolated to these settings.

## 5. Conclusions

In conclusion, this retrospective cohort study of more than 60,000 individuals from general practices in Germany revealed that PTSD was not significantly associated with incident CLBP in the overall population. However, the relationship was positive and significant in people aged >60 years, suggesting that the older population may be particularly vulnerable to the deleterious effects of PTSD on the musculoskeletal system and more particularly on the low back. Finally, further research of longitudinal nature is warranted before any firm conclusion is drawn.

## Figures and Tables

**Table 1:** Characteristics of the study patients after 1:1 matching.

Variable	PTSD (*n* = 30,332)	No PTSD (*n* = 30,332)	*p*-Value ^1^
*Age at baseline (in years)*			
Mean (SD)	46.0 (16.7)	46.0 (16.8)	0.997
18–30	21.3	21.4	0.811
31–40	18.8	18.5	
41–50	20.7	20.6	
51–60	20.0	20.2	
>60	19.2	19.3	
*Sex*			
Female	62.7	62.7	0.973
Male	37.3	37.3	
Duration of follow-up in years, mean (SD)	5.2 (3.9)	5.7 (4.0)	<0.001
Mean number of consultations during the follow-up, mean (SD)	8.8 (8.5)	8.8 (8.5)	0.968
*Conditions documented within 12 months prior to or at the index date*			
Hypertension	26.5	27.8	<0.001
Disorders of the thyroid gland	23.0	23.1	0.637
Disorders of lipoprotein metabolism and other lipidemias	18.8	17.2	<0.001
Gastritis and duodenitis	15.5	12.9	<0.001
Injury of unspecified body region	13.4	11.6	<0.001
Diabetes mellitus	12.9	13.5	0.037
Chronic sinusitis	11.1	8.9	<0.001
Sleep disorders	11.0	5.2	<0.001
Other and unspecified dermatitis	8.8	9.0	0.512
Joint disorders, not elsewhere classified	8.5	8.0	0.037
Gastro-esophageal reflux disease	7.8	7.1	<0.001
Asthma	7.7	8.1	0.079
Overweight and obesity	7.1	7.2	0.777
Vasomotor and allergic rhinitis	7.0	7.1	0.568
Other enthesopathies	7.0	6.1	<0.001
Migraine	6.4	5.5	<0.001
Cancer	6.0	5.6	0.046
Chronic ischemic heart disease	5.7	6.3	0.005
Shoulder lesions	5.7	5.1	0.001
Other disorders of muscle	5.5	4.8	<0.001
Varicose veins of lower extremities	5.0	5.1	0.326

Abbreviations: PTSD, post-traumatic stress disorder; SD, standard deviation. Data are percentages unless otherwise specified. ^1^ *p*-values were obtained using the Wilcoxon signed-rank test for continuous variables, the McNemar test for categorical variables with two categories, and the Stuart–Maxwell test for categorical variables with more than two categories.

**Table 2:** Association between post-traumatic stress disorder and the cumulative incidence of chronic low back pain in adults followed in general practices in Germany.

Population	Incidence per 1000 Person-Years in Patients with PTSD	Incidence per 1000 Person-Years in Patients without PTSD	HR (95% CI)	*p*-Value
Overall	10.7	9.7	1.07 (0.99–1.15)	0.081
*Age at baseline (in years)*				
18–30	6.1	7.3	0.83 (0.68–1.01)	0.061
31–40	10.1	10.1	0.98 (0.83–1.16)	0.806
41–50	12.6	11.5	1.09 (0.95–1.25)	0.246
51–60	12.5	10.7	1.13 (0.97–1.31)	0.108
>60	11.7	9.0	1.24 (1.05–1.46)	0.011
*Sex*				
Female	10.1	9.1	1.09 (0.99–1.20)	0.066
Male	11.6	11.0	1.03 (0.92–1.15)	0.633

Abbreviations: PTSD, post-traumatic stress disorder; HR, hazard ratio; CI, confidence interval. Cox regression models were adjusted for the conditions listed in Table 1 (i.e., hypertension, disorders of the thyroid gland, disorders of lipoprotein metabolism and other lipidemias, gastritis and duodenitis, injury of unspecified body region, diabetes mellitus, chronic sinusitis, sleep disorders, other and unspecified dermatitis, joint disorders, not elsewhere classified, gastro-esophageal reflux disease, asthma, overweight and obesity, vasomotor and allergic rhinitis, other enthesopathies, migraine, cancer, chronic ischemic heart disease, shoulder lesions, other disorders of muscle, and varicose veins of lower extremities).

## Data Availability

Data and code used in this study are available from the corresponding author upon reasonable request.
